# Ethics of emerging infectious disease outbreak responses: Using Ebola virus disease as a case study of limited resource allocation

**DOI:** 10.1371/journal.pone.0246320

**Published:** 2021-02-02

**Authors:** Ariadne A. Nichol, Annick Antierens

**Affiliations:** 1 Center for Biomedical Ethics, Stanford University School of Medicine, Stanford, California, United States of America; 2 Médecins Sans Frontières, Brussels, Belgium; Division of Clinical Research, UNITED STATES

## Abstract

Emerging infectious diseases such as Ebola Virus Disease (EVD), Nipah Virus Encephalitis and Lassa fever pose significant epidemic threats. Responses to emerging infectious disease outbreaks frequently occur in resource-constrained regions and under high pressure to quickly contain the outbreak prior to potential spread. As seen in the 2020 EVD outbreaks in the Democratic Republic of Congo and the current COVID-19 pandemic, there is a continued need to evaluate and address the ethical challenges that arise in the high stakes environment of an emerging infectious disease outbreak response. The research presented here provides analysis of the ethical challenges with regard to allocation of limited resources, particularly experimental therapeutics, using the 2013–2016 EVD outbreak in West Africa as a case study. In-depth semi-structured interviews were conducted with senior healthcare personnel (n = 16) from international humanitarian aid organizations intimately engaged in the 2013–2016 EVD outbreak response in West Africa. Interviews were recorded in private setting, transcribed, and iteratively coded using grounded theory methodology. A majority of respondents indicated a clear propensity to adopt an ethical framework of guiding principles for international responses to emerging infectious disease outbreaks. Respondents agreed that prioritization of frontline workers’ access to experimental therapeutics was warranted based on a principle of reciprocity. There was widespread acceptance of adaptive trial designs and greater trial transparency in providing access to experimental therapeutics. Many respondents also emphasized the importance of community engagement in limited resource allocation scheme design and culturally appropriate informed consent procedures. The study results inform a potential ethical framework of guiding principles based on the interview participants’ insights to be adopted by international response organizations and their healthcare workers in the face of allocating limited resources such as experimental therapeutics in future emerging infectious disease outbreaks to ease the moral burden of individual healthcare providers.

## Introduction

Emerging infectious disease (EID) threats are increasing due to a complex array of factors including changing land use and more frequent regional and global travel [1, 2]. Today, a cluster of cases can become thousands in a matter of weeks if an individual decides to travel, unaware of their infection status or in pursuit of medical attention. Strategies for bolstering preparedness for decision-making in an outbreak response should be devised within an ethical frame, or else resulting decisions could harm affected communities and exacerbate existing health disparities.

Prior to 2020, the 2013–2016 Ebola Virus Disease (EVD) outbreak in West Africa was considered one of the most threatening emerging infectious disease epidemics of modern history. The outbreak resulted in 28,616 cases and 11,310 deaths, posing a serious global threat [3–5]. The public health and medical interventions implemented to try to combat the outbreak raised ethical dilemmas related to issues of justice, respect for persons, and beneficence. From military-imposed quarantines to informed consent processes, the EVD outbreak demonstrated many cases in which critical moral decisions were resolved under harsh field conditions and extreme time-constraints [3, 6–9]. Currently, another EVD outbreak has occurred in Equateur Province of the Democratic Republic of Congo and COVID-19 caused by SARS-CoV-2 is widespread across the globe, highlighting the urgent need to more carefully examine and address the ethics of emerging infectious disease outbreak responses, particularly those surrounding decisions regarding allocation of limited resources.

The qualitative study presented here works with a data set from extensive interviews of senior healthcare personnel (n = 16) from several organizations that were heavily involved in the 2013–2016 EVD outbreak response. The interviewed healthcare workers acted in a variety of capacities, both on the ground during the outbreak and at international headquarters, therefore the study encompasses a variety of perspectives. The results of this study inform a potential ethical framework based on interviewed responders’ insights that can be adopted by healthcare workers in the face of emerging infectious disease outbreaks. Topics such as monitored use of experimental products, implementation of clinical trials, informed consent and community engagement are covered within the framework. EID outbreak preparedness response strategies are needed; however, the strategies must be carefully considered from an ethics standpoint, or else resulting decisions can harm affected communities, increase existing health disparities and exacerbate the outbreak by prolonging the duration and scale of it.

## Methods

### Typing & justification of study methodology

The research objectives were to elucidate an understanding of allocation decisions in the context of a high-profile EID response in resource-constrained regions. A qualitative study design was selected to provide the broad and open inquiry necessary to derive meaningful conclusions [10–13]. A semi-structured interview process was selected to ensure consistency in the interviews, while maintaining flexibility in hearing first-hand subjective experiences [14].

### Population sample & selection

Inclusion criteria for the study were English-speaking individuals over 18 years of age with extensive work experience for any international humanitarian medical organization directly involved in the 2013–2016 EVD outbreak response. This study was conducted one year after the 2013–2016 EVD outbreak ended.

Eligible prospective participants were recruited through direct, online email solicitation. Forty emails were sent and 25 affirmative voluntary responses were received. Given scheduling opportunities and time constraints on the part of the research team, 16 respondents were interviewed. Respondents were selected based on having made substantial contributions to the West Africa 2013–16 Ebola response, worked in a variety of roles and included personnel across three different international response organizations. The participant demographics included 6 males and 10 females aged 40–65. Eight participants were field doctors or nurses, deployed through their organizations to provide direct clinical care to affected communities, 7 participants occupied leadership roles in emergency medical coordination and technical advisement and 1 participant served on the legal team at the headquarters of their organization. The range of roles provided an opportunity to capture a variety of different experiences and perspectives. Participants with clinical field experience during the 2013–2016 EVD outbreak had worked in Guinea, Liberia and Sierra Leone. Length of field deployments ranged from 2–12 weeks, with all clinicians having participated in multiple deployments. Some participants also had robust leadership experience in decisions relating to use of experimental therapeutics in the outbreak.

Throughout the study, IRB approved procedures were followed to protect the confidentiality of the research participants. All documents were approved by the IRB at Stanford University. This included the participation solicitation email, the informed consent form, the background information document (S1 File), the pre-interview questionnaire (S2 File), and the interview guide (S3 File) each participant received.

### Data collection

All participants were given an informational document explaining the nature of the research (S1 File) and an opportunity to ask questions. After receiving the information, the consent form was obtained from all participants before interviews commenced and participants were requested to fill in the pre-interview questionnaire (S2 File). The interviews were conducted in-person in order to allow for clarity in interpreting non-verbal cues and ambiguous responses [15]. All interviews were conducted in private to ensure confidentiality and audio taped to enhance descriptive validity of the data. An interview guide (S3 File) was used to limit interviewer bias and seek standardization of question delivery across interviews [13]. The interviewer asked participants open-ended questions regarding limited resources in outbreak responses, allocation decisions, informed consent experiences, and moral obligations. A hypothetical scenario was included on how to allocate drug therapies if only a limited supply existed. Participants were asked to verbally walk the researcher through their decision-making processes. Interviews ranged between 1 to 1.5 hours in length. No remuneration was provided.

### Data organization & analysis

The data were stored on Box to maintain file security and confidentiality. Interviews were transcribed verbatim. Transcripts (3135–9359 words) were cleaned to remove any identifiers and then indexed. An iterative process of coding based on grounded theory was used to ensure trustworthiness of the emergent categories [11]. Analytic memos were written after each step of the coding process in order to make additional insights and connections [11].

Open coding was employed in a first pass aimed at analyzing the textual content [11], along with several external codes (e.g. beneficence, do no harm) derived from a seminal source on the principles of biomedical ethics [16]. The study codebook is provided in S1 Table. Axial coding was employed in a second pass to identify and cluster conceptual categories (S2 Table). Examples of codes included in the category of values were ‘do no harm,’ ‘fairness,’ and ‘solidarity.’ These codes demonstrate the moral principles as articulated by the participants. Data saturation was reached when each category was explored in depth and no new insights resulted.

## Results and discussion

### General challenges of the outbreak response

The results of the study captured a range of insights in regard to ethical issues that can arise during an EID outbreak based on the personal accounts of the interviewed participants (see S1 Table for sample quotations). A large majority of respondents (81%) felt that the 2013–2016 West Africa EVD outbreak presented a particularly difficult response situation. Many (75%) also expressed feelings of frustration and hopelessness regarding the response efforts. Several participants identified that the course of EVD was particularly difficult for them to observe even as clinicians who have encountered many diseases, such as one clinician who described the experience as “very brutal.” Roughly half (56%) mentioned how difficult they found it to work under extreme time-pressured conditions. The intensity of the outbreak is well described by one participant as “more like a war than a normal outbreak.”

### Ethical decisions regarding allocation of clinical staff time and physical resources in the field

Many of the decisions with a moral dimension specifically involved issues of limited resource allocation. The core element of an EVD outbreak response is to identify cases and quickly isolate them in order to prevent further person-to-person transmission, while providing medical care. Patient care was mostly provided in rapidly built Ebola Treatment Centers (ETCs). Limited number of beds led to difficult admission decisions. Lack of trained staff resulted in dilemmas in time-management for experienced staff regarding prioritizing time spent training personnel versus time spent directly managing patient care. To provide context, the requirements of wearing highly restrictive personal protective equipment (PPE) while caring for patients severely limited time spent in the ETC ward. For some PPE used in the treatment centers, only approximately 60 minutes can be spent in full PPE before having to exit to minimize risk of overheating and dehydration, straining the health care staff to define priorities in terms of which patients to care for and how comprehensive the care for each patient could be.

Roughly two thirds of participants (63%) were able to recall specific instances where they felt unsure of whether they had made the ‘right’ or moral decision regarding time or resource allocation. In hindsight, most stood by their decisions made at the time, acknowledging the high-pressured, time-sensitive environment they were in and the lack of other feasible options. Instances of such value-laden decisions made for powerful accounts of lived experience within the midst of an EID outbreak, where competing priorities of advancing public health measures and caring for individual patients proved difficult to balance.

One particularly difficult moral dilemma was whether to continue to admit EVD cases who had nowhere else to go, into an ETC already at capacity with regard to beds and number of patients that could be reasonably cared for. To quote one participant: “We started to have ambulances and minibuses of eight to ten patients coming in, and we didn’t have the space. I never considered that we would not admit those patients… but in hindsight I should have probably looked after my staff more before thinking of patients.”

Another participant’s account highlighted an example of a dilemma that arose from choosing between providing robust patient care and ensuring staff safety: “I made sure everyone had a water bottle and some food. And then that was it… then I focused on the IPC [Infection Prevention and Control]… if I would not have done that, doctors would have not been able to come and give better care a day later.” This participant allocated her time to first provide the absolute basic needs of food and water to patients, and then spend the remainder of her day focused on implementing infection prevention and control measures. Her reasoning was to prioritize setting up a safe environment for healthcare workers so that the following day additional staff could come into the ETC to provide quality care for the patients. This choice between staff safety and providing individual patient care came up several times in participants’ examples of moral dilemmas they faced on the ground during the 2013–2016 EVD outbreak in West Africa.

### Allocation of experimental therapeutics available in limited quantities

#### Use of experimental therapies via compassionate use or clinical trials

The topic of how to allocate limited medical resources in a humanitarian response is not novel; however, a unique aspect of an emerging infectious disease outbreak such as EVD is the associated high case fatality rate in conjunction with development and testing of experimental therapeutics. Experimental therapeutic interventions can be provided via compassionate use and/or clinical trials. Often clinical trials take considerable time to establish, have exclusion criteria and utilize a placebo control arm. Compassionate use differs from clinical trials because it is implemented on a case-by-case basis for individuals after determining that the probable risk to the patient from the investigational therapeutic is not greater than the probable risk from the disease [17, 18]. A World Health Organization (WHO) Advisory Panel recommended monitored emergency use of unregistered and investigational interventions (MEURI) during the 2013–2016 EVD outbreak [19]. MEURI must be enacted in conjunction with necessary supportive treatment, creating a more significant monitoring standard [20].

All participants supported the utilization of MEURI when appropriate. Several participants specified that MEURI would be appropriate when establishment of clinical trials would be lengthy or were not possible to set up. One participant described MEURI as a “gap filling measure” that should be done in a way that the observational data could be in some way aggregated with trial data. Another participant felt that one monoclonal antibody cocktail being tested at the time in a clinical trial, was not used enough through MEURI to ensure access to vulnerable populations such as pregnant women who were not eligible to enroll in the trial: “The ZMapp [a monoclonal antibody cocktail] was available for trial and for emergency use, and I feel we didn’t use it enough as emergency use.”

Roughly half of the participants (56%) supported prioritizing clinical trials over MEURI or other forms of compassionate use. Of those supporting clinical trials, 66% of respondents did so with the caveat of not supporting the implementation of a standard randomized control trial (RCT), where treated cases are compared with a control group without specific intervention. The participants cited the high case fatality rate associated with EVD when vocalizing this caveat. Participants felt that placing patients into a non-intervention control group in an RCT trial would be unethical, since patients would only receive supportive care which would result in very low chance of survival. One participant’s comments captured this sentiment: “I personally would disagree to randomize against placebo. I know that it lacks scientific evidence otherwise but in this case because of the high case fatality rate… it’s not ethical.” Another participant compared the control group as an equivalent to a “death sentence” for patients.

Other reasons against utilizing an RCT structure included impracticality of the trial design and the potential negative community perception of randomization to a control arm or an experimental arm within an RCT structure: “No one disagrees that a randomized control trial is scientifically the best way to get data. But, I think that each situation is different in terms of what is practical to do.” Participants who voiced concern felt that the local communities had not been involved enough in shaping the outbreak response and that community engagement efforts should be prioritized when selecting a trial design to ensure it is culturally acceptable and well understood.

#### Use of adaptive clinical trial designs

When asked about trial design, all participants (100%) advocated for the use of adaptive designs in trial structure. Concerns over the ethical justification of non-adaptive trial procedures were raised several times, and participants called for the procedures of any trial to be “ethically sound from the beginning.” The design of the trials should be applicable to the nature of disease and for the population where trial recruitment will occur. None of the participants disagreed with the notion that an RCT is the gold standard for trial design, as exemplified in the participant response: "So what I think we really need is a repertoire of different trial designs, recognizing that the RCT is the ideal design, and then coming down to other adaptive designs when necessary, and then tailoring our particular design to a particulate situation." One participant explained that they felt trial protocols should be finalized with the community, and that the first beneficiary should be the community where the trials are established because that population is bearing the risks of participating in such trials to help advance scientific understanding: “The design of the trials needs to be ethically acceptable for the disease and for the population. The whole system. If it is not working, you have to stop it immediately. If it is working, you have to finalize it with the community, and the first beneficiary will be this community where you are making the trials.”

#### Ethical considerations of therapeutics access relative to vulnerable populations

Pregnant or lactating women or children are often excluded from phase I and phase II safety trials of potential new therapeutics or vaccines, frequently leading to decreased access to promising treatments. 25% of participants had concerns regarding the exclusion of pregnant women and children in clinical trials. The absence of evidence disadvantages pregnant women in that the lack of inclusion in trials then prevents pregnant women from accessing experimental therapeutics because of the lack of safety data for use in pregnant women: “Excluding pregnant and all of that, it’s always the same trick. The absence of evidence plays against these people in the end because then you’re prevented from doing anything to them.” Such concerns about the cyclical argument that results in exclusion of pregnant women from accessing experimental therapeutics have also been raised by others [21].

One participant stated that it was not ethical to exclude children under the age of 6 on the basis of “a purely theoretical supposition that the therapeutic might be dangerous for their health,” justifying the use of a therapeutic by the fact that EVD is deadlier in children than in adults. Children under the age of 4, infected with Ebola virus, have a 80–90% case fatality rate [22]. Several participants stressed that MEURI would be appropriate when vulnerable populations are excluded from clinical trials due to not meeting the inclusion criteria (e.g. pregnant women and/or children).

#### Prioritization of access to limited therapeutics based on role in outbreak

One constraint of both clinical trials or use of MEURI for experimental therapeutics is that they are often available in limited quantities. The challenge then becomes how to allocate the limited resource in an equitable manner. Only two participants suggested first-come first-serve as a fair scheme of allocation. Interestingly, many of the other participants disagreed with the notion of first-come first-serve and felt it would be “unethical” to use such a scheme that appeared to be lottery-like because it would prove impossible to justify to the affected communities.

The majority of participants (75%) supported the prioritization of healthcare workers, particularly frontline workers directly involved in the response, in accessing experimental therapeutics either through clinical trial participation or via MEURI. Participants justified the response by citing the risks incurred by frontline workers by providing essential services and the utility of restoring health to those capable of saving other lives in the future. For example, one participant stated: “Every Ebola survivor is not equal and the one that can go back onto the front lines has a utility above that of a construction worker in the setting of an outbreak.” As another example, a participant justified the prioritization in this manner: “Those guys are risking their lives. They deserve to have special treatment. And also in an egocentric approach, you need healthcare workers in good health to save other lives. You have to privilege them.” To provide context, 8% of Liberia’s healthcare workers died as a result of EVD during the 2013–2016 outbreak [23].

Within the group that supported this type of prioritization of frontline workers, 25% of the participants directly mentioned the principle of reciprocity, which captures how the risks undertaken by healthcare workers in providing a societal benefit should be reciprocal to the benefits that society can provide by prioritizing healthcare workers in this public health context. Also, many of those who viewed prioritization of healthcare workers as fair, also supported the view that national healthcare staff should receive the same prioritization in access to limited experimental therapeutics as the international medical responders. One participant cited the sense of solidarity between the healthcare staff as justifying this lack of difference between the healthcare workers: "I have never experienced such a sense of solidarity. It’s very special. When you’ve been together under this PPE and you’ve been confronted with people dying on a daily basis… You share the emotion. You share the risk."

#### Access to limited therapeutics based on who would benefit most

Another favored approach involved prioritizing allocation of experimental therapeutics to those that would potentially benefit most from the therapeutic, based on clinical prognosis (44%). High levels of virus detected in early blood samples taken from symptomatic EVD cases correlates with high case fatalities. For context, virus is quantitated by polymerase chain reaction (PCR) and measured in CT units from 1–40, which provide an inverse proxy for the amount of virus present. Participants advocated for not providing experimental therapeutics to cases with high viral loads, thereby ensuring therapeutic supply for those cases with lower viral loads that would be likely to benefit the most from treatment. For example, a participant described favoring this approach based on CT values: “Clearly, there was literature on the CT, as a proxy for the viral load, and there’s evidence that with a CT much lower than 18, the survival chances were very, very low. So I would base my decision on this kind of evidence- chance of survival- not to waste any product.”

#### Prioritizing access to limited therapeutic based on social value

Roughly a third of the participants (31%) supported including social value criterion when they explained what they would consider when distributing a limited experimental therapeutic. Several participants used the example of prioritizing mothers since they occupy caregiver roles in their communities or prioritizing high-profile community leaders within communities. However, a minority of the participants (19%) strongly opposed favoring mothers as caregivers, citing that this kind of prioritization unfairly assigned more moral worth to a person with children. This minority felt it would be “a slippery slope” to argue that caregivers have more duties, or duties with greater moral worth assigned by society, than women without children. For example, one participant stated: “What would you do for a person without kids? Do they have less duties or something? I think it’s a slippery slope to put a worth on a person…”

### Engaging with affected communities

Many participants emphasized the need for community involvement in whatever allocation scheme was to be agreed upon. Several felt that community perception of the decisions made, contributed to or negatively impacted the success of the overall outbreak response. Rumors and misconceptions surrounding the international response posed significant challenges in ensuring affected individuals and families entered ETCs and understood their options and in safeguarding frontline workers involved. For example, one rumor recounted by participants was that international responders were spreading EVD when they were decontaminating areas such as infected individuals’ homes or local hospitals: “The problem is when you’re not affected directly and the stakes are so high, it’s very easy to trick people into believing stuff. It was amazing the rumors that were going around like we were spreading the disease when we were decontaminating.”

Issues of transparency and additional safeguards towards vulnerable populations were also raised. Multiple participants felt that selection criteria for the allocation schema of experimental therapeutics should be “extremely transparent” and that local communities should be involved in the decision making process. One participant also addressed the potential ramifications of inaction to individuals and affected communities when weighing the risks and benefits of providing an experimental therapeutic through clinical trials or emergency use: “You would have to have an appreciation for what’s the risk and what’s the cost to the person of inaction.”

### Challenges of informed consent

Several participants identified informed consent procedures for participating in clinical trials or receiving emergency use of experimental therapeutics as potential areas for improvement, to ensure that vulnerable patients are consented in a culturally appropriate manner. Two participants specifically brought up the issue of therapeutic misconception, where individuals do not understand the concept of a clinical research trial and inappropriately believe they will receive therapy. Another stated that in some communities, written consent is not appropriate, and verbal consent should instead be practiced.

### Need for an ethical framework

When asked about utilizing an ethical framework, there was a clear propensity to adopt an ethical framework of guiding principles (88%) for emerging infectious diseases like EVD. Several reasons to establish guiding principles for future EID outbreaks were commonly cited including the complex nature of the decision-making processes regarding clinical trial design which decrease the likelihood of rapid scientific advancement and the lack of standardization across international humanitarian responses.

Two participants directly mentioned that an ethical framework would be comforting to the healthcare workers in the field, in serving as a source for guiding principles: “It could help the person make the decision. And it would make people more comfortable with themselves in making a decision.” They strongly advocated for formation of principles to ease healthcare worker’s personal moral burdens when facing these situations by turning towards collective decision-making, where no single person has to make these kinds of decisions seemingly on their own: “It can never be one person’s decision. It’s always a collective decision.”

Interestingly, 25% of the participants felt that clinicians in the field were too close to individual patients and communities to be able to make sound decisions on how to distribute the experimental therapeutics without bias. For example, two participants articulated that they would have made an “emotional decision” if they were asked to provide an experimental therapeutic to a colleague who had contracted EVD.

In terms of which basic principles they wanted reflected in the framework for allocation, participants brought up many of the fundamental biomedical research ethics principles, including but not limited to: respect for persons; protection of dependent or vulnerable populations; do no harm; and a principle of justice. A principle of equity was also discussed several times in the form of wanting to ensure post-trial access to local affected populations and in offering those ineligible for trials access to experimental therapeutics via MEURI. One participant described respect for persons as “seeing people as autonomous agents with a right to self-determine.”

Interestingly, several participants (19%) described their personal sense of right and wrong by using anecdotes to articulate their moral reasoning. For example, one participant used an anecdote of walking along the beach and seeing two people drowning: “If I’m walking on the beach and I’m the only person on the beach and I see two people drowning, I don’t say: ‘well I’m not going to save one of them because I’m not going to save both of them.’” They would not take the approach of ignoring both drowning individuals because they cannot save both of them, instead they would save one person and then advocate for “more lifeguards on the beach to be able to save all of them.” Overall, the respondents indicated a strong desire to maintain high ethical standards during challenging and dynamic outbreak responses like the 2013–2016 EVD outbreak.

### Proposed framework for allocation of experimental therapies

Several important themes emerged from this study including the prioritization of frontline workers’ access to experimental therapeutics when available in limited quantities, widespread acceptance of adaptive clinical trial designs, improved clinical trial transparency and greater engagement with the local affected communities. An overwhelming majority of responders were in favor of the development of an ethical framework of guiding principles to benefit those in the field response in the next EID outbreak, especially when allocating limited experimental therapeutics.

Others have also called for new guidelines for more clearly defining boundaries between clinical trials and compassionate use [24]. The World Health Organization (WHO) had issued ethics criteria for the use of unregistered interventions for EVD; however, important ethical questions remained unanswered [19, 25]. While EVD outbreak ethical considerations have been discussed earlier in specific contexts (e.g. individual countries), it was thought that relevant insights informing a potential framework would prove useful, and could be implementable in the field in resource-constrained regions tackling EID outbreaks specifically with high case fatality [26–29]. Analysis of the insights shared in the interviews of the international healthcare workers involved in the 2013–2016 EVD outbreak response provided the basis for such an ethical framework (Table 1).

**Table 1:** Proposed framework of guiding principles for preparedness and implementation by international response organizations for EID outbreaks.

**Community Engagement**	▪ Promote collaboration and open dialogue by creating streams of bi-lateral information between all stakeholders (e.g. medical response coordinators, researchers, local frontline responders, community members)
	▪ Incorporate community insights into decision-making processes by consultation of the community leaders, community members, and local clinical expertise
	▪ Reflect the context-specific cultural values and norms
	▪ Encourage transparency on the part of responders to ensure the legitimacy of the response
	▪ Build trust and foster relationships between communities and responders for potential responses in the future
	▪ Reduce rumors that result in infected persons hiding out and not receiving medical care by actively working to alleviate fear in communities
**Experimental Therapeutic Interventions**	▪ Provide therapeutics to beneficiaries in monitored settings
	▪ Minimize harms from potential side-effects
	▪ Offer MEURI to vulnerable populations excluded from clinical research trial eligibility criteria (e.g. pregnant women, children under 5 years of age)
	▪ Prioritize frontline healthcare workers
	▪ Do not prioritize expatriated healthcare workers over local frontline responders
**Clinical Trial Designs**	▪ Prioritize establishment of clinical trials
	▪ Offer MEURI prior to the start of clinical trials and through post-trial access
	▪ Consider the possibility of alternative trial designs to the standard randomized controlled trial (RCT)
	▪ Include stoppage rules to minimize potential harm
	▪ Employ interim analysis to promote on-going evaluation and reflection
**Informed Consent**	▪ Respect individual autonomy
	▪ Minimize coercion and undue influence
	▪ Protect vulnerable populations
	▪ Foster understanding of clinical trials and reduce therapeutic misconception
	▪ Utilize appropriate medical terms, matching the level of fluency in scientific vocabulary
	▪ Create culturally meaningful consent and consider alternatives to written forms based on community feedback and local expertise

This framework derived from responses from individuals heavily involved in outbreak responses in the largest Ebola Virus Disease outbreak in history (West Africa 2013–2016), may complement guidelines developed by international ethics experts, including those in the recent U.S. National Academies of Sciences, Engineering, and Medicine report [30, 31]. Such frameworks and guidelines should prove very useful for those working at international humanitarian medical organizations, to inform strategy and preparedness for future responses to EIDs, specifically for EIDs with high case fatality rates highlighted in a WHO priority list of top emerging pathogens, which includes EVD [32, 33]. These frameworks and guidelines will not remain static but should be continually updated in the light of new knowledge and tailored to specific EID outbreaks. For instance, reduction in case fatality rate estimates may influence acceptance of RCT trial designs utilizing a non-intervention control arm and alter decisions on who may benefit most from treatment when experimental therapeutics are in limited supply.

The analysis presented here is relevant to the current EVD outbreak in western Democratic Republic of Congo [34] and many of the recommendations are applicable to the current COVID-19 outbreak response [35]. Community mistrust has continued to plague subsequent Ebola outbreak responses and impacted response efforts [36, 37].

Recent steps in the right direction include updated MEURI guidelines and modified Ebola therapeutics trial designs [38–40]. For instance, in response to concerns expressed regarding RCT design with a standard of care only control arm, the recent PALM trial of Ebola experimental therapeutics used an interventional control arm, where three additional therapeutics (REGN-EB3, MAb114 and Remdesivir) were compared to ZMapp as the control arm [39, 41, 42]. A pre-existing ethical framework of guiding principles can continue to accelerate access and trial design and improve community engagement on limited resource allocation, reducing rushed decisions and improving the speed and quality of outbreak responses.

## Supporting information

S1 File Background information document. (DOCX)

S2 File Pre-interview questionaire. (DOCX)

S3 File Interview guide. (DOCX)

S1 Table Codebook. (DOCX)

S2 Table Mapping of open codes to axial codes. (DOCX)
