# Supplementary material for: Ethics of emerging infectious disease outbreak responses: Using Ebola virus disease as a case study of limited resource allocation
Source: PLoS One. 2021 Feb 2;16(2):e0246320. doi: 10.1371/journal.pone.0246320 (PMC7853513; doi:10.1371/journal.pone.0246320)
Supplement: S1 File — (DOCX) [file pone.0246320.s001.docx]

**Background Information Document**

**Purpose of the Study**

- To develop a more nuanced understanding of the ethical challenges involved the just distribution of limited resources, specifically experimental drug therapies, vaccines, and biomaterial samples, within an emerging infectious disease outbreak setting

**Specific Objectives**

- To explore the ethical issues surrounding the use of experimental drug therapeutics and vaccines
- To explore the challenges involved in data sharing among different stakeholders
- To explore the ethical implications of setting research priorities
- To explore how informed consent of the beneficiaries is obtained and perceived

**Study Methodology**

- This is a qualitative study using semi-structured interviews with health care workers involved who were involved in the 2013-2016 Ebola outbreak

**Limited Resources**

- Can be defined as physical resources (hospital beds, medical supplies, therapeutics, vaccines, samples, etc.), personnel resources (number of staff), training resources (educational materials), and opportunity resources (research, gaining further knowledge, etc.)
- For this specific study, interested in experimental drug therapeutics and vaccines as physical resources in terms of both supply and access, in addition to sample data as an opportunity resource in terms of conducting further research

**Relevant Ethical Principles**

- **Beneficence**
  - Risk/benefit ratios
- **Justice**
  - Fairness
  - Equity
  - Reciprocity
  - Solidarity
  - Transparency
  - Accountability
- **Respect for Persons**
  - Autonomy
  - Informed consent
  - Vulnerable populations

**Experimental Therapeutics and Vaccines**

- Includes immunomodulators, anti-viral drugs, mono-clonal antibody cocktails, small inhibitory RNA, and convalescent blood plasma transfusions, and vaccines that have unproven safety and efficacy but show potential in relevant *in vitro* and animal models

**Different Intervention Contexts**

- Experimental therapeutics and vaccines can be implemented via compassionate use, monitored emergency use of unregistered and experimental interventions (MEURI), or clinical trials
