# Supplementary material for: Ethics of emerging infectious disease outbreak responses: Using Ebola virus disease as a case study of limited resource allocation
Source: PLoS One. 2021 Feb 2;16(2):e0246320. doi: 10.1371/journal.pone.0246320 (PMC7853513; doi:10.1371/journal.pone.0246320)
Supplement: S2 File — (DOCX) [file pone.0246320.s002.docx]

**Pre-Interview Questionnaire (1min)**

*(Only very brief answers are necessary or simple yes/no)*

| How long have you worked at your organization?  What is your role within your organization? |
| --- |
| Do you have prior field experience?  Have you been involved in any Ebola outbreak?  If yes, in what capacity and for how long? |
| Have you been involved in discussions/decisions regarding the use of experimental products for Ebola? |
| Are you familiar with the concept of biobanking and data sharing platforms? |
