# Supplementary material for: Ethics of emerging infectious disease outbreak responses: Using Ebola virus disease as a case study of limited resource allocation
Source: PLoS One. 2021 Feb 2;16(2):e0246320. doi: 10.1371/journal.pone.0246320 (PMC7853513; doi:10.1371/journal.pone.0246320)
Supplement: S3 File — (DOCX) [file pone.0246320.s003.docx]

**Interview Guide**

1. Could you briefly describe what its like to work within an outbreak response with high case fatality rates, such as the 2013-2016 Ebola outbreak?

- As a medical practitioner, how you balance patient’s interests with broader public health interests during an emergency response?
- Did you ever have an experience where you felt rushed to make some kind of ethical decision during the response and when you look back, feel unclear as to whether you made the ‘right’ decision? If so, please explain.

2. In an outbreak response situation where access to experimental drug therapies or vaccines was limited, what was (or would be) your decision making process?

- How did you (or would you) go about deciding how to handle the available resources in terms of just distribution among the patients/beneficiaries?
- Who were (or would be) the relevant stakeholders involved in the particular situation?
- Do you think there were (or would be) differences in priorities held by the different stakeholders (patients, government health authorities, clinicians, medical researchers, international outbreak response partners etc.)?
- Who ended up (or would end up) receiving the drug therapy or vaccine?
- Were you (or would you be) satisfied with the resulting distribution in terms of equitability?

3. When you were involved in the 2013-2016 Ebola Virus Disease (EVD) outbreak, what was the decision making process regarding the distribution of experimental drug therapies or vaccines to patients/beneficiaries?

- What is your opinion on this decision making process?
- What decision making process would you suggest for the next similar situation?

4. In terms of weighing the benefits and risks in the allocation of experimental therapeutic products, do you feel that clinical trials should be prioritized over individual emergency use?

- Under which conditions should individual emergency use be implemented?
- Should health care workers have preferential access to therapeutic products by either emergency use or participation in clinical trials? What about frontline workers?^1^
- What kind of decision-making process should be followed in order to evaluate which therapeutic products will be included in clinical trials?
- What kind of clinical trial design should be implemented within this kind of outbreak response?

5. Do you think having an ethical framework in place for allocating limited resources like experimental therapeutics and biomaterial samples before a disease outbreak response would be of value?

- If so, what would be the best way to develop such a framework?
- Which ethical principles do you believe should be afforded more or less weight?
