# Supplementary material for: Ethics of emerging infectious disease outbreak responses: Using Ebola virus disease as a case study of limited resource allocation
Source: PLoS One. 2021 Feb 2;16(2):e0246320. doi: 10.1371/journal.pone.0246320 (PMC7853513; doi:10.1371/journal.pone.0246320)
Supplement: S1 Table — (DOCX) [file pone.0246320.s004.docx]

**Codebook**

| **Open Codes** | **Definitions** | **Example Quotation 1** | **Example Quotation 2** |
| --- | --- | --- | --- |
| **Limited resources** | Physical resources or valuable resources (e.g. time or clinical expertise) that are limited in quantity or ease of access to some degree | "Even if you use it [an experimental therapeutic] as compassionate use, you should collect the data because the opportunity to do research is so limited" | "It would be extremely difficult to implement… unless we had twice the staff" |
| **Patient-doctor interactions** | Descriptions of interactions between physicians and patients, such as when the physician is providing supportive care or treatment, obtaining informed consent, giving explanations, or talking with the patients and their immediate family members | "I had to go around feeding and giving drinks to my patients, which I did, but I was in a rush" | "When you ask them to consent, you’ve already asked them a lot of questions, they arrive in an environment that is really stressful… And I found that some of the first versions were really too difficult for the patient to understand" |
| **Challenge** | A situation or undertaking that is particularly difficult (either physically or mentally) and requires a degree of strength or skill to overcome | "It was more like a war than a normal outbreak" | "It is not easy to work in this kind of setting, where in fact you have more chance of a patient dying than a patient living" |
| **Community engagement** | Partnering with community members to foster dialogue, engage in discussions, and make collective decisions | “The design of the trials needs to be ethically acceptable for the disease and for the population. The whole system. If it is not working, you have to stop it immediately. If it is working, you have to finalize it with the community, and the first beneficiary will be this community where you are making the trials” | "The community part, was always a big part of the intervention so people go into the community and explain the disease and explain how you get infected and explain how there is an ETC and explain what is done in a ETC" |
| **Informed consent** | Permission freely given by a patient (or designated guardian) to a doctor for a treatment or procedure, fully aware of the potential risks and benefits | "It must be freely given, it cannot be tie to the provision of any services, it can’t be bundled with other forms of consent, it must be a positive, unambiguous act" | "It very, very difficult to have an actual informed consent in those circumstances. The best that I can say is that we informed the patient... again because of the case fatality rate, most people will agree" |
| **Time constraint** | A limitation or restriction in the amount of time available to accomplish an objective | “It was mainly this feeling when you enter in a ETC with patients, where you know you have limited time, and then straight away you make already these decisions in your mind like ‘what are the priority patients?” | "I would not delay. If the protocols were ready I would start the trial" |
| **Moral relativism** | A philosophical position that moral or ethical propositions do not reflect objective and/or universal moral truths, but instead make claims relative to social, cultural, historical or personal circumstances | "If they were going to say first the men, then the women, then I might have a problem with that. Then you get into moral relativism sort of stuff there" | "I don’t want to defend cultural relativism...I don’t know what is ethics, although I use the word very often. I don’t know if it’s universal, or if it’s relative to culture or time. I think the truth is in between" |
| **Protecting vulnerable populations** | Ensuring individual rights within vulnerable populations, where people may not be able to give consent or may be coerced into participation of research | "...refraining from contributing to new inequities and taking advantage of low-resource countries or vulnerable populations by conducting research inexpensively or in less regulated countries" | “It’s not ethical to exclude children under 6 on the basis of a purely theoretical supposition that the vaccination might be dangerous for their health. If they get Ebola, they have more than 50% chance of dying. This is also true for pregnant women” |
| **Benefit** | An advantage or good gained from something | "I need to understand who would be benefited more, which kinds of patients, if they are children, CT below 15… I don’t want to spoil the drug. I want to use the drug where it has the best possibility of working" | "They should do something that has some benefit. In the long run, do you want to put $5 in everyone’s pocket or give them better access to a nurse when they really need it?" |
| **Risk** | A danger that could cause harm or loss | "I think you’re willing to take more risk and tolerate adverse things" | "...very specific teaching that you have to do before the person says ‘yes I want it or no.’ I think they need to be offered the choice with as much information as we have based on what would be potential risks to them and the amount of people those things have happened to" |
| **Do no harm** | Actions that do not cause harm to others, also known as non-maleficence | "...do no harm" | "…be sure that it’s not harmful and not worse for him [the patient] to use" |
| **Beneficence** | Actions that are done for the benefit of others | "...maximizing possible benefits and minimizing possible harms" | "I think if there’s a clear expectation that there will be a benefit for the patient and that the potential side effects will not kill the patient" |
| **Need for guidance** | Wanting advice or information from an authority or expert on how to resolve a problem or difficulty | "We felt lost. No one was guiding us" | "They took the decision not to give the experimental treatment, which I think was the right decision considering there was no framework" |
| **Finding a balance** | Comparing the value of one thing to another in order to reach a condition in which the different elements are equal or in the correct proportions | "I made sure everyone had a water bottle and some food. And then that was it. I didn’t give any medical care as such. And then I focused on the IPC... If I would not have done that, doctors would have not been able to come and give better care a day later… In Ebola I think it’s always this balance" | "It’s a risk benefit balance" |
| **Bringing stakeholders together** | When several relevant stakeholders (patients, physicians, ethics board members, local government officials, pharmaceutical companies) come together to share their perspectives with one another and address their concerns/goals | "We have this in-house experimental therapeutics advisory board that I was part of, that would be a stakeholder, or a place where the stakeholders come together, depending on how you look at it" | "...have a bilateral partnership with the ministry of health and the government" |
| **Knowledge gap** | A lack of scientific knowledge on a particular topic | "He [the patient] might be less educated than you, he might know less science than you, but be transparent" | "I think most of the people don't understand what exactly it is because it’s written in dry and sometimes too scientific language" |
| **Preparation** | The action or process of getting ready for future epidemic events via developing plans | "…have the protocols ready and all the stakeholders agree on the criteria, before the next outbreak" | "The procedures of the trial need to be ethically sound from the beginning" |
| **Perception** | A way of understanding or interpreting something such as an act, event, or decision | "If the perception is going to result in, two days after the event, an incredibly strong reaction from the community, rightly or wrongly, that could even be violent and endanger people, you can’t say ‘well, even though that perception or reaction is wrong I’m going to completely discount it.’ So I think it [the perception] has to be part of the decision, but not the whole of the decision" | "I thought when the president of Liberia decided to go with incineration with the burials because there were too many bodies around and nobody had the capacity to conduct the burials… I was convinced that the people would refuse, but they didn’t. In the first month, they accepted the cremation. In Africa, I know more or less because of years of work in Africa, and I saw that my cultural knowledge, my perception was biased in this situation" |
| **Transparency** | Acting in a way that is free from pretense or deceit, characterized by visibility or accessibility of information | "And I’m very transparent. I’m the first one to say that it’s really not to save them, but that it’s really to protect their family because they will be a risk" | “It’s very important for me that you set up selection criteria and that you’re extremely transparent about it” |
| **Rumors** | A statement or report without known authority for its truth | "But the rumors are almost always there that we are researchers there just to steal their organs" | "In West Africa, it was amazing the rumors that were going around like we were spreading the disease when we were decontaminating" |
| **Utility** | The amount of satisfaction or advantage gained from the outcome that results from a particular decision | "I have 20 doses of drug X and 6 patients. What would be the best way to make use of them? The tension that always comes up in humanitarian affairs is justice vs. utility. The problem is that utilitarian calculations are very hard to make on the fly. You’re not going to know what 20 patients you’re going to have to come up with the weighted decision of the downstream effects" | "I think the utilitarian criteria would not work in this situation precisely because we do not know the utility. It’s experimental. You still have big questions about risks and benefits, so what is the utility?" |
| **Placing responsibility** | Designating a particular burden of obligation and accountability onto a person or organization | "I think the responsibilities when something goes wrong are different. I mean if you a regulator and you agree on a use of something without having any idea of how it works, I think… I do think everyone has a different perspective. As a clinician with a patient, I would want to try anything to save the patient. But if I’m a regulator in the country maybe I’m not so confident that this never-heard-of drug should be used on the patient in the country" | "I would take the decision out of their hands [clinicians on the ground] completely" |
| **Fairness** | A principle of social justice/equity in valuing the absence of socially unjust or unfair burdens | "...fair distribution of benefits and risks of the research" | "It’s not fair to randomize on an individual basis, and it’s practically impossible. It would be like a sophist choice" |
| **Equality** | A principle of having people be treated as equal with regard to status, rights, and opportunities | "For me, people are the same, unless there is a clinical reason. The other one that you asked about types of people, like clinicians yes or no, I don’t know. That would be a funny one for me, as my whole principle of equality of people is too much the same" | "You have to fight against these inequalities in a feasible way and at the level you have the capacity to. It depends on the intuition, on the context, on the resources, on a lot of factors. But you have to fight for this" |
| **Respect for persons** | To recognize the worth of a person or thing and hold them in regard | "...respect for people as autonomous agents and that is most commonly embodied in patient consent" | “…seeing people as autonomous agents with a right to self-determine” |
| **Moral worth** | The moral weight something holds | "What would you do for a person without kids? Do they have less duties or something? I think it’s a slippery slope to put a worth on a person" | "Every Ebola survivor is not equal and the one that can go back onto the front lines has a utility above that of a construction worker in the setting of an outbreak" |
| **Historical controls** | A trial design that utilizes historical data as a control | "For other diseases you may not have several thousand historic controls like we now have for Ebola" | "Offer the treatment to everybody, and compare that to maybe some historical data" |
| **RCT use** | A randomized control research trial with a treatment arm and a non-interventional control arm | "There are people advocating for the fact that strict adherence to an RCT would get you the quickest answer" | "I personally would disagree to randomize against placebo. I know that it lacks scientific evidence otherwise but in this case because of the high case fatality rate… it’s not ethical" |
| **Prioritization** | Establishment of the importance or the urgency of actions that are necessary or people that are necessary | “I made sure everyone had a water bottle and some food. And then that was it... And then I focused on the IPC [Infection Prevention and Control]… if I would not have done that, doctors would have not been able to come and give better care a day later” | “Women and children on a sinking boat are always a priority, but in fact if you have a kid who can swim and you have a guy who can’t swim, you should give the priority to the guy who can’t swim, you know? So for each disease, identify the priority groups… not ‘who’ they are, but how to define them” |
| **Solidarity** | A feeling of unity among individuals with a common interest | "To control an outbreak, everyone needs to get together. It’s not just clinical work" | "I have never experienced such a sense of solidarity. It’s very special. When you’ve been together under this PPE and you’ve been confronted with people dying on a daily basis... You share the emotion. You share the risk" |
| **High case-fatality rate** | A disease with a death rate of over 50% | "Some other people would argue with a disease with so little to lose, with 90% mortality, this balance may shift" | "I personally would disagree to randomize against placebo. I know that it lacks scientific evidence otherwise but in this case because of the high case fatality rate, I would have to agree that it’s not ethical" |
| **Professional obligations** | Duties that stem from the nature of one's profession, such as helping patients for doctors | "I got a lot of negative feedback from external doctors that thought I was a very crappy clinician because I focused on IPC and not the patients" | "If we do not do our job properly, healthy people get sick, communities could get sicker, and the burden of the disease could become much bigger" |
| **Moral obligations** | A duty which one owes and ought to perform but is not legally binding to fulfill | "You should probably give the people, through the representatives of the communities, a say in what they would like" | "But I think that unless something really terrible happens to pregnant women that we know about it, it should be offered to them- but not just given, really make sure people understand what they are getting" |
| **Hopelessness** | A feeling or state of despair associated with a lack of hope | "I’m a clinician, so I’m used to see people dying but not in this way. This is very brutal" | "It is quite heavy on your staff, on your self, to have such a high case fatality rate because you do whatever you can, but people are dying" |
| **Autonomy** | The quality or state of being self-governing | "…seeing people as autonomous agents with a right to self-determine" | "You [as a pregnant woman] don’t even have the choice to do something about it yourself. You cannot decide ‘yes, please put me in the trial because I value my life more than… because I have five kids at home and I don’t want them to die of Ebola’" |
| **Frustration** | A feeling or state of anger and disappointment in response to opposition or perceived lack of achieving something | "I think it’s the biggest frustration to go to that outbreak and not even establish the preventive measures" | "As a clinician, it’s a very frustrating experience I would say" |
| **Safety concerns** | Concerns regarding how safe the conditions for providing care are for frontline workers, other staff members, etc. | “We started to have ambulances and minibuses of eight to ten patients coming in, and we didn’t have the space. I never considered that we would not admit those patients… but in hindsight I should have probably looked after my staff more before thinking of patients” | "...but then the challenge also of safety of the healthcare worker. I have done some things that I probably shouldn’t have done in terms of my own safety, trying to give a level of care that I thought was close to the ideal as I could make it, and then realizing after that that was probably not a wise thing to do" |
| **Compassionate use** | Use of an experimental therapeutic implemented on a case-by-case basis | "...we can sort of MEURI our way along until we can officially start the trial, or something like that. It might be something you would do as a gap filling measure. And you would like to do it in a way that the data could be in some way aggregated with the trial data” | "We have used compassionate use... in this case of the newborn, where we were sure that normally they would never survive. We say okay let’s go for this last chance thing via MEURI" |
| **Clinical trials** | Use of an experimental therapeutic through clinical trials | "At least with a trial we will have some answers regarding this new treatment or new vaccine" | "I think clinical trials, the way they are designed nowadays are definitely not adapted to this kind of epidemic" |
| **Experimental therapeutics** | Therapeutics including immunomodulators, anti-viral drugs, mono-clonal antibody cocktails, small inhibitory RNA, convalescent blood plasma transfusions, and vaccines that have unproven safety and efficacy but show potential in relevant in vitro and animal models | "Well to my knowledge, all of the expats were flown back abroad, so they had access to the best available experimental products... Personally, I don’t think it is ethically acceptable, if there’s no possibility to… no, well let’s put it like this: I don’t think it is ethically acceptable to give privilege to an expat compared to national staff" | "I think with these experimental interventions you should try to try to involve the patient and the family as much as possible" |
| **Adaptive trial designs** | Adaptive designs for clinical trials to provide evidence of the effectiveness and safety of a therapeutic, as interpreted by respondent | "If you have a disease as you say, with a high case fatality rate and short duration in time and also that the outbreak will normally be limited in time, like Ebola, like SARS, I think making a case control trial is not adapted to those specific situations. I don’t say that case control is not useful. It’s useful for different problems, but in those specific diseases like SARS and Ebola, we need to find another way to give hope and save lives in a quicker way than a classical case control" | "So what I think we really need is a repertoire of different trial designs, recognizing that the RCT is the ideal design, and then coming down to other adaptive designs when necessary, and then tailoring our particular design to a particulate situation" |
| **Uncertainty** | A feeling or state of being uncertain or experiencing indecisiveness when making choices (the opposite of black and white decisions) | "Things are always a sliding scale, so I think you would say that there are certain situations where it’ll be near to impossible to ask consent and there are the others where you could build in more discussion or some kind of consultation, depending on what stage the patient is at, depending on the exact conditions. You know like it’s not always a black or white, it’s always a grey scale" | "I do definitely believe there is a point of no return. And I think this is one we take into account, but it’s not proven again so it takes very, very clinical judgment, clinical feeling... And I feel like my team has a very tough time to decide on this moment because it’s not black and white" |
| **Reciprocity** | A principle in the public health context that grants workers privileges, and in a reciprocal way expects them to perform certain functions that benefit society and saves lives | "I think they [frontline workers] deserve some sort of priority… it’s called the principle of reciprocity" | "Someone who’s there who took a voluntary risk. Of course they can get infected in their homes just like everybody else, but nevertheless there was a voluntary engagement with risk and I think that carries a moral weight to it" |
| **Lottery allocation** | Allocating therapeutics via a lottery-like system (e.g. first-come first-serve basis) | "In such a disease, or such a case fatality rate, I think it is very hard to put people in a lottery. It’s not humane or ethically acceptable" | "I would say perhaps, a lottery, and I know most of the people would be absolutely against the lottery, but that has to be discussed beforehand" |
