# Supplementary material for: Ethics of emerging infectious disease outbreak responses: Using Ebola virus disease as a case study of limited resource allocation
Source: PLoS One. 2021 Feb 2;16(2):e0246320. doi: 10.1371/journal.pone.0246320 (PMC7853513; doi:10.1371/journal.pone.0246320)
Supplement: S2 Table — (DOCX) [file pone.0246320.s005.docx]

**Mapping of Open Codes to Axial Codes**

| Open Codes | Axial Codes |
| --- | --- |
| Challenge | Descriptions of outbreak response |
| Patient-doctor interactions |  |
| Frustration | Emotions |
| Hopelessness |  |
| Uncertainty |  |
| Safety concerns | Challenges of limited resources |
| Limited resources |  |
| Time constraint |  |
| Challenge |  |
| Uncertainty |  |
| Compassionate use | Use of experimental therapeutics |
| Clinical trials |  |
| Experimental therapeutics |  |
| Adaptive trial designs |  |
| RCT |  |
| Historical controls |  |
| Protecting vulnerable populations | Views on how to allocate experimental therapeutics |
| Prioritization |  |
| Lottery allocation |  |
| Reciprocity |  |
| Benefit |  |
| Risk |  |
| Moral worth |  |
| Utility |  |
| Moral relativism |  |
| Community engagement | Engaging communities |
| Bringing stakeholders together |  |
| Perception |  |
| Transparency |  |
| Rumors |  |
| Knowledge gap | Informed consent challenges |
| Informed consent |  |
| Do no harm | Values |
| Beneficence |  |
| Fairness |  |
| Solidarity |  |
| Respect for persons |  |
| Autonomy |  |
| Equality |  |
| Professional obligations | Obligations |
| Moral obligations |  |
| Need for guidance | Need for an ethical framework |
| Finding a balance |  |
| Placing responsibility |  |
